# Editorial: Advances in ocular surface disease

**DOI:** 10.3389/fmed.2023.1357275

**Published:** 2024-01-09

**Authors:** Hong Qi, Jin Yuan, Kyung Chul Yoon, Hong Liang

**Affiliations:** ^1^Department of Ophthalmology, Peking University Third Hospital, Beijing, China; ^2^Beijing Key Laboratory of Restoration of Damaged Ocular Nerve, Beijing, China; ^3^State Key Laboratory of Ophthalmology, Zhongshan Ophthalmic Center, Sun Yat-sen University, Guangzhou, China; ^4^Department of Ophthalmology, Chonnam National University Medical School and Hospital, Gwangju, Republic of Korea; ^5^Department of Ophthalmology III, CHNO des Quinze-Vingts, Paris, France; ^6^Sorbonne Université, INSERM, CNRS, Institut de la Vision, IHU FOReSIGHT, Paris, France

**Keywords:** ocular graft-vs.-host disease, inflammation, diabetes, lacrimal gland, pterygium

The ocular surface comprises the conjunctiva, cornea, lacrimal gland, and related eyelid structures (1). Ocular surface disease encompasses a range of disorders impacting its normal structure and function, leading to symptomatic discomfort, visual disturbances, and tear film instability. Common types include dry eye disease, keratitis, conjunctivitis, and pterygium. Systemic conditions like diabetes and graft-vs.-host disease can trigger severe neuropathy and inflammation in ocular surface tissues. Our understanding of ocular surface disease has significantly advanced in recent decades through notable progress in basic and clinical research.

To showcase this, we initiated the Research Topic “*Advances in ocular surface disease*”, and published five original research articles and one review focusing on etiology, diagnosis, and treatment.

Sleep disorders, part of lifestyle challenges, are linked to increased severity of dry eye symptoms and clinical signs, as well as keratoconus grade (2). Obstructive Sleep Apnea Syndrome (OSAS) involves repeated upper airway obstruction during sleep, leading to arousal with or without oxygen desaturation, posing a significant risk for multi-organ dysfunction. Hao et al. found higher ocular surface disease index (OSDI) and partial blink rates in OSAS patients compared to healthy volunteers. Severe OSAS patients exhibited higher corneal fluorescein staining scores and lower tear break-up time, underscoring the need for heightened ocular surface care in OSAS patients.

Uncontrolled dietary glucose intake and obesity are closely linked to diabetes mellitus (DM). While diabetic retinopathy is well-known, DM also profoundly affects the cornea, leading to diabetic neurotrophic keratopathy, which is estimated to affect 47–64% of diabetic patients (3). Although neurotrophic keratopathy is a major cause of corneal morbidity, the link between diabetes mellitus and keratoconus remains debated. Zhu et al. suggested that higher fasting glucose might genetically reduce the risk of keratoconus by performing Mendelian randomization analysis. The potential mechanism involves heightened collagen cross-linking of cornea in diabetes mellitus patients, indicating that a rational sugar diet strategy might help curbing keratoconus.

Beyond metabolic disorders like diabetes mellitus (DM), various systemic immune conditions pose a threat to ocular surface homeostasis. Ocular graft-vs.-host disease (oGVHD), prevalent in the chronic stage of GVHD post-allogeneic hematopoietic stem cell transplantation, is primarily triggered by an excessive effector T cell immune response in the lacrimal gland, conjunctiva, and cornea, resulting in refractory and prolonged dry eye (4). Based on a series of studies on clinical manifestations, diagnostic biomarkers of oGVHD (4, 5), Shen et al. from Hong Jing's team developed a user-friendly diagnostic model, combining CFS, Schirmer's test score without anesthesia, and conjunctival score, promising reliable screening for chronic oGVHD with an AUC of 0.945. Lacrimal gland fibrosis takes the core position in oGVHD-related DED development. Lin et al. reviewed and summarized advances in clinically examining the lacrimal gland, emphasizing functional assessments focusing on tear quality and quantity, such as TBUT, tear meniscus height, and the tear ferning test, providing crucial diagnostic information for ocular surface diseases impacting the lacrimal gland.

Ocular surface mucosal systems are specialized for eye lubrication and immune defense (6). Cornea is immune-privileged to promise a clear vision, but instead it becomes vulnerable during ocular surface inflammation. In immune-mediated diseases, from corneal transplant rejection to Stevens-Johnson Syndrome, anti-inflammatory agents like corticosteroid derivatives play a crucial role in therapy, preventing corneal opacity and ulcers from the inflammatory cascade. Prolonged corticosteroid use is known to elevate intraocular pressure (IOP). Hence, anti-inflammatory alternatives that don't affect IOP are preferred. RCI001, containing 8-oxo-20-deoxyguanosine (8-oxo-dG), inhibits Rac1 and the NLRP3 inflammasome, making it a novel drug candidate for inflammatory eye diseases. Kim et al. observed that mice treated with RCI001 showed no significant increase in IOP until week 5, suggesting its safety in treating inflammatory ocular surface diseases.

Pterygium is a prevalent ocular surface disease with a global incidence of up to 12%, and involves degeneration and hypertrophy of the bulbar conjunctiva and subconjunctival tissue, extending into the cornea. This progression affects aesthetics and corneal morphology, increases wavefront aberrations, poses irregular astigmatism of the cornea and decrease visual quality. Zhang et al. compared corneal densitometry (CD) values in eyes with pterygium and unaffected eyes using Pentacam. Results showed that pterygium-affected eyes exhibited elevated CD values, especially in the anterior and central layers, aligning with the understanding that pterygium primarily invades Descemet's membrane and the cornea's superficial stromal layer.

Ocular surface diseases disrupt local mucosal homeostasis, causing tear film instability, corneal and conjunctival damage, posing a significant threat to comfort and clear vision. In conclusion, this Research Topic enhanced our understanding of the etiology, diagnosis, and treatment of ocular surface diseases in some aspect, with a specific focus on dry eye disease, keratoconus, ocular graft-vs-host disease (oGVHD), and pterygium.

## Author contributions

HQ: Writing—original draft, Writing—review & editing. JY: Writing—review & editing. KY: Writing—review & editing. HL: Writing—review & editing.
